# The planarian regeneration transcriptome reveals a shared but temporally shifted regulatory program between opposing head and tail scenarios

**DOI:** 10.1186/1471-2164-14-797

**Published:** 2013-11-16

**Authors:** Damian Kao, Daniel Felix, Aziz Aboobaker

**Affiliations:** School of Life Sciences, University of Nottingham, University Park, Nottingham, NG7 2RD UK; Fundación CNIC Carlos III- Centro Nacional de Investigaciones Cardiovasculares, Melchor Fernández Almagro, 3, Madrid, Código Postal 28029 Spain; Department of Zoology, University of Oxford, The Tinbergen Building, South Parks Road, Oxford, OX1 3PS UK

**Keywords:** Regeneration, PREP homeodomain, Planarian, Transcriptome, Stem cell, RNAseq

## Abstract

**Background:**

Planarians can regenerate entire animals from a small fragment of the body. The regenerating fragment is able to create new tissues and remodel existing tissues to form a complete animal. Thus different fragments with very different starting components eventually converge on the same solution. In this study, we performed an extensive RNA-seq time-course on regenerating head and tail fragments to observe the differences and similarities of the transcriptional landscape between head and tail fragments during regeneration.

**Results:**

We have consolidated existing transcriptomic data for *S. mediterranea* to generate a high confidence set of transcripts for use in genome wide expression studies. We performed a RNA-seq time-course on regenerating head and tail fragments from 0 hours to 3 days. We found that the transcriptome profiles of head and tail regeneration were very different at the start of regeneration; however, an unexpected convergence of transcriptional profiles occurred at 48 hours when head and tail fragments are still morphologically distinct. By comparing differentially expressed transcripts at various time-points, we revealed that this divergence/convergence pattern is caused by a shared regulatory program that runs early in heads and later in tails.

Additionally, we also performed RNA-seq on *smed-prep*(RNAi) tail fragments which ultimately fail to regenerate anterior structures. We find the gene regulation program in response to *smed-prep*(RNAi) to display the opposite regulatory trend compared to the previously mentioned share regulatory program during regeneration. Using annotation data and comparative approaches, we also identified a set of approximately 4,800 triclad specific transcripts that were enriched amongst the genes displaying differential expression during the regeneration time-course.

**Conclusion:**

The regeneration transcriptome of head and tail regeneration provides us with a rich resource for investigating the global expression changes that occurs during regeneration. We show that very different regenerative scenarios utilize a shared core regenerative program. Furthermore, our consolidated transcriptome and annotations allowed us to identity triclad specific transcripts that are enriched within this core regulatory program. Our data support the hypothesis that both conserved aspects of animal developmental programs and recent evolutionarily innovations work in concert to control regeneration.

**Electronic supplementary material:**

The online version of this article (doi:10.1186/1471-2164-14-797) contains supplementary material, which is available to authorized users.

## Background

Understanding how we might replace damaged and diseased tissue through the use of stem cell based therapies is an important goal for biomedical science. Despite natural occurrences that occur across the Animal Kingdom and work in a growing number of systems, regeneration is still poorly understood. Model systems are starting to shed light on the molecular processes that orchestrate various regenerative phenomena [1, 2]. Among the various systems, planarians have a distinct advantage of being able to regenerate entire animals from small starting fragments [3–8]. To what extent this is due to novel mechanisms unique to planarians is unknown. The simple anatomy and highly accessible adult stem cell system of planarian flatworms make it a high value model system from which we can hope to form valuable paradigms for how regeneration is controlled.

Regenerative potential in these animals is dependent on a population of cycling pluripotent adult stem cells present throughout the parenchyma, except in the area in front of the photoreceptors and the region of the pharynx [3–7]. On wounding or amputation these adult stem cells undergo two characteristic peaks of cell division that produce stem cell progeny and can subsequently differentiate to replace missing or damaged tissue [9]. The amenability of planarians, and in particular the planarian *Schmidtea mediterranea* to functional genomic approaches in combination with advances in sequencing technology has produced several gene expression and transcriptomic studies on head regeneration [10], wounding [9], and neoblast dynamics [11–14].

Planarians are able to reconstitute the full adult body plan from very different starting scenarios. For example tail stump pieces will regenerate a new head, head pieces a new tail and trunk fragments both a head and a tail. In addition to all these potentially different regenerative scenarios, the planarian must also rescale and remodel the morphology of pre-existing tissues and organs to fit the size of the animals and to ensure sufficient functional integration [15]. Thus from very different beginnings all fragments converge to the same end point. This means that any starting fragment contains the information required to reconstitute the whole adult body. While we already know a little about some of the key events in this process, for example the signalling pathways that are required to ensure the correct polarity along the different axes of regenerating pieces, we still lack an understanding of how these events fit together globally [7, 8, 16–19].

In this study we have amalgamated existing transcriptomes [10, 13, 14, 20, 21] to provide an improved resource as a service to the research community. This exercise includes re-annotation of transcripts, removal of likely chimeric transcripts and characterisation of transcripts that code for proteins novel to the phylum Platyhelminthes and/or the intensely studied Triclad group of planarians.

Using this new meta-transcriptome assembly we looked to investigate the potential for genome wide expression analysis for understanding differences and similarities in the regulatory program that underpins different regenerative scenarios. We investigated head and tail regeneration of the planarian *Schmidtea mediterranea* from 0 to 72 hours after amputation. Using this wealth of data, we were able to describe patterns of gene expression levels during regeneration across the whole transcriptome and perform comparisons between regeneration time-points and scenarios. This allowed us to describe the transcriptional changes that reflect key regulatory transitions during the regenerative process.

We found that head regeneration (head fragment regenerating tail) and tail regeneration (tail fragment regenerating head) transcriptomes initially reflect the differences in cellular content at the beginning of regeneration. They then diverge further over the first 12 hours of regeneration. However, we observed an unexpected convergence of expression profiles by 48 hours of regeneration between these two contrasting scenarios. This divergent/convergent pattern was underpinned by a core battery of more than 5,000 genes that are regulated in the same manner during between 6-12 hours of head regeneration and 36-48 hours of tail regeneration.

Both to internally validate our data at the genome wide level and to further define those genes that can be associated specifically with anterior regeneration we performed RNA-seq in the background of the well characterised *Smed-prep(RNAi)* phenotype, which specifically results in the loss of anterior structures [22]. This allowed the identification of putative direct and indirect targets of *Smed-prep* which were enriched among those genes we found to be involved in the shared regulatory transition.

From blast annotations against selected species, we were able to define lists of genes that are potentially unique to *S. mediterranea*, unique within the tricladida [23], and unique to the phylum Platyhelminthes. We found that differentially expressed transcripts during regeneration were enriched for triclad specific transcripts. These transcripts are potentially involved in novel mechanisms underpinning potent regenerative capacity, and suggest that as has been recently suggested in urodeles, some important aspects of regeneration may be lineage specific [24, 25]. Our data provide new insight into the regulatory logic of regeneration, and act as a reference point against which to advance our understanding of the regulatory control of regeneration.

## Results and discussion

### Consolidation of the available transcriptome data

There are currently five independently assembled *S mediterran*ea transcriptomes with sufficient read depth and coverage to have aspirations to providing whole transcriptome coverage [10, 13, 14, 20, 21]. It is unclear in the literature to what extent, if at all, later assemblies have used data from earlier sources. We decided against a reassembly from the raw sequencing data that went into the 5 independent assemblies due to the varying error profiles of the raw data from different library preparation and sequencing chemistry. In addition, not all data was readily available and/or described fully in the repositories. We gathered the available transcriptome data from the 5 relevant publications (we refer to transcriptome datasets by the group leader’s last name) along with the available EST datasets and performed a consolidation of the transcripts. We chose to include only 5 of the 6 available transcriptomes because the dataset provided by Abril et al. contained a high number of contigs (~192,000) suggesting a highly fragmented assembly [26]. We did not want to introduce more variability into the consolidation.

The consolidation process seeks to retain a high confidence set of transcripts, resolve transcript fusion events, and retain transcripts with the longest open reading frames. We first clustered transcripts from all 6 datasets by sequence similarity using CAP3 assembler [27]. Each assembled contig can be represented as a cluster with contributing transcripts from one of the 6 data sources. We kept only clusters that had transcripts from at least 2 different sources to ensure a high confidence set of transcripts resulting in 23,802 clusters. We then removed potential fusion transcripts from each cluster by analysing the position of top blast hits along the cluster length to complete proteome sets. Removal of potential fusion transcripts split 441 clusters into 1,014 clusters. To retain the sequence with the longest ORF for each cluster, we took the transcript or CAP3 contig with the longest ORF in each cluster.

To make sure we are including known *S. mediterrane*a transcripts that might not be in the 5 transcriptomes due to low expression, we blasted the consolidated transcripts to 179 known *S. mediterranea* mRNA sequences. 16 transcripts were not found in the consolidated transcriptome including genes with very low expression levels or very localised expression patterns (e.g. *Smed-wnt-1, Smed-noggin-like 4, Smed-noggin-like 6*) and many neuropeptide pro-hormones with restricted expression patterns [16, 28, 29]. These data are indicative that genes with expression restricted to small populations of cells may well have escaped current sequencing efforts.

**Figure 1:**
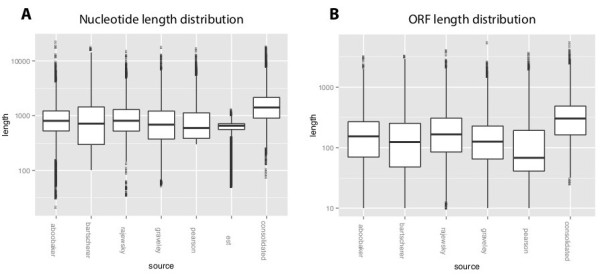
**Length and open reading frame length distribution of consolidated transcriptome vs individual transcriptomes. A)** Boxplots showing the distribution of transcript lengths in base pairs among the input data sets (5 transcriptomes and 1 EST dataset) and the consolidated transcriptome **B)** Boxplot showing the distribution of open reading frame lengths among the input data (5 transcriptomes) and the consolidated transcriptome.

A detailed description of the process is available as a Additional files 1, 2, 3, 4, 5, 6, 7, 8 containing all relevant scripts and details. The consolidated transcriptome contains 23,545 transcripts of which, 2,859 are unmodified transcripts taken from one of the transcriptome sources and the remaining 20,686 transcripts are assembled CAP3 contigs. A comparison of average transcript length and N50 length to the 5 transcriptomes shows a substantial length increase with respect to both transcript length and open reading frame (ORF) length (Figure 1A and B). We also assessed transcriptome coverage by blasting each transcriptome to the Core Eukaryotic Gene Mapping Approach (CEGMA) database [30] which contains a core set of genes found in a wide range of eukaryotic organisms. The consolidated transcriptome had hits to 449 CEGMA genes out of a possible 458 and a significantly larger ortholog hit ratio of 0.93 compared to the 5 transcriptomes (Table 1).

**Table 1:** **CEGMA hits and ortholog hit ratios of consolidated transcriptome vs individual transcriptomes**

	Consolidated	Aboobaker	Bartscherer	Rajewsky	Graveley	Pearson	EST
**CEGMA hits**	449	443	435	444	303	439	410
**CEGMA ortholog hit ratio**	0.93	0.88	0.82	0.88	0.73	0.88	0.68

Overall our analyses show that a simple consolidation of the extant published data provides an improved high confidence *S. mediterranea* transcriptome with respect to representation, total length and coding potential. This will be of significance for future genome wide expression analyses exploiting this important model system.

### A gene expression time-course of anterior and posterior regeneration

Planarian regeneration is able to confidently restore whole individuals, with all organs scaled to the correct size from any starting piece [15]. Thus from very different beginnings, the same end result is obtained. In the first instance we wished to understand how this process is reflected in whole transcriptome gene expression changes. One would expect early expression profiles to be very different depending on the cell and tissue contents, for example brain and neural tissues in the head versus gut tissues in the tail. The expression profiles should eventually converge as the missing tissues are regenerated.

Our goal was to describe these trajectories from different starting scenarios to obtain an overview of which genes are differentially expressed during the first 72 hours of regeneration. We hypothesised that differentially expressed genes would represent the unique regulatory solutions each regenerative scenario uses to arrive at the reconstitution of a whole animal. To this end we performed transcriptome sequencing on regenerating head and tail fragments at 0, 6, 12, 24, 36, 48 and 72 hours after amputation.

In total, 514,384,160 reads were mapped to the transcriptome across replicate samples at each regeneration time-point in each of the two scenarios. On average, the correlation between replicate samples was 0.99. While having 2 replicates for each time-point/fragment is not ideal for modelling the variance encountered in RNA-seq, our sample preparation of including multiple individuals (20 fragments in each library) does offer some variance stabilization through biological averaging. Having more replicates would add more resolution to our individual transcript expression profiles, but for the purpose of observing global trends in expression, we resorted to statistical optimizations of filtering our dataset more stringently and setting a higher adjusted p-value threshold.

**Figure 2:**
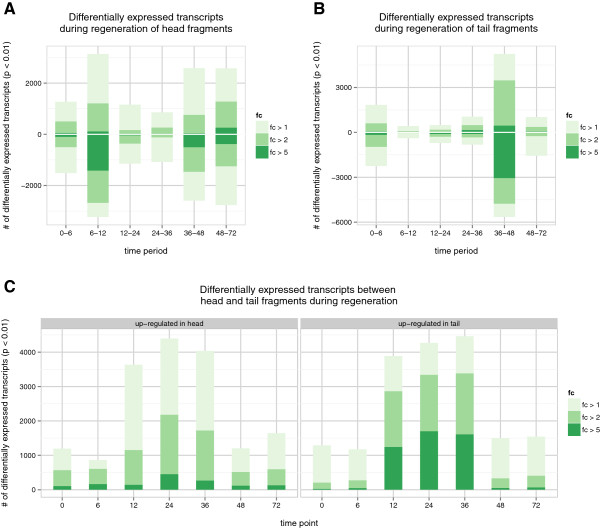
**Differential expression during head/tail regeneration and head vs tail regeneration.** Differential expression performed with EdgeR was done on consecutive time-points during head and tail regeneration (0-6, 6-12, 12-24.) and also between head and tail expression profiles at the same time-points. **A)** Head regeneration time-course showing the number of differentially expressed transcripts at p-value < 0.01 and 3 different fold changes. Positive numbers represent the number of transcripts that are up-regulated between two time-points and negative numbers represents down-regulation. **B)** Tail regeneration time-course showing the number of differentially expressed transcripts at p-value < 0.01 and 3 different fold changes. **C)** Differential expression between head and tail fragments during regeneration at p-value of 0.01 and 3 different fold changes.

We filtered our raw count data for transcripts that had less than 20 reads mapping in all libraries, leaving us with 15,423 transcripts for differential expression analysis. We performed differential expression analysis with EdgeR [31] on pairs of consecutive time-points (0-6 hours, 6-12 hours, 12-24 hours…etc) and also on pairs of fragments at the same time-point (0 hour head vs 0 hour tail, 6 hour head vs 6 hour tail…etc). The significance threshold for differential expression was set at p-value of 0.01 (Figure 2A-D).

The largest number of differentially expressed transcripts during head and tail regeneration are 3,228 transcripts down-regulated from 6-12 hours in heads and 5,646 transcripts down-regulated from 36-48 hours in tails (Figure 2A and B). We will refer to lists of differentially expressed transcripts by their abbreviated fragment type and time-period. For example, transcripts down-regulated from 6 to 12 hours in head fragments will be abbreviated as H6-12-down. We also observed that between fragments there is considerable increase in differentially up-regulated transcripts in both head and tail fragments between 12 and 36 hours. (Figure 2C). This increase hints at a divergence of expression profiles between head and tail regeneration starting at 12 hours and ending after 36 hours. This divergence may be representative of a differential program utilized by head and tail fragments. At higher fold-changes, there are also more transcripts up-regulated in tail fragments compared to head fragments between 12 and 36 hours suggesting that tail fragments undergoes a more drastic expression regulation during the divergence than head fragments. A possible reason for this may be that tail fragments need to regenerate a brain which contains a rich population of genes and isoforms.

Overall our data set describes the transcriptome of head and tail fragments during the first 72 hours of regeneration. This time-course data reflects the processes of regenerating new tissues in addition to remodelling existing tissue since whole fragments were used instead of just the regenerating blastema. This dataset presents a valuable resource for data mining the transcriptional behaviour of planarians genes during regeneration.

### Head and tail fragment enriched transcripts implicates genes involved in early anterior and posterior regeneration

As a simple validation of our expression data, we looked for head and tail enriched transcripts by comparing head and tail fragments at 0 hours, immediately after amputation. We were able to find many known anterior markers (*arrestin*[32], *opsin*[33], *tyrosinase*[34]*, eyes absent*[35], *wnt2-1*[16], *otxA*[36], *six1*[35], *prep*[22]) and posterior markers (*wnt11-1, wnt11-2*[37], *AbdBa*[38], *hox-D*[36], *axin*[39]) enriched in head and tail piecess respectively (Tables 2 and 3). We also find many membrane voltage gated channels and neurotransmitter receptors involved in nervous system function, metalloproteinases, and several homeobox genes (*NK-1, cut-like, lim, orthopedia, vsx-1*) in head enriched transcripts. We will refer to head and tail enriched transcripts at the beginning of regeneration as F-head and F-tail (Table 4).

**Table 2:** **Head enriched transcripts**

id	Blast hit	Species	Fold-change	Citation
OX_Smed_1.0.02775	Arrestin, beta 2b	Danio rerio	6193.32778476	Nakazawa et al [32]
OX_Smed_1.0.12568	Opsin mRNA, partial cds	Schmidtea mediterranea	97.2862560359	Alvarado et al [33]
OX_Smed_1.0.01148	Tyrosinase mRNA, complete cds	Schmidtea mediterranea	72.546116361	Lapain et al [34]
OX_Smed_1.0.10865	Glutamate receptor, AMPA, putative	Schistosoma mansoni	15.3249112099	Agata et al [40]
OX_Smed_1.0.20161	Smed-NDK mRNA, partial cds	Schmidtea mediterranea	12.1805738905	Agata et al [40]
OX_Smed_1.0.06957	Secreted frizzled protein-like protein (SFRP-a) mRNA, partial cds	Schmidtea mediterranea	11.8424203893	Gurly et al [41]
OX_Smed_1.0.06840	G protein alpha subunit (Gpas) mRNA, partial cds	Schmidtea mediterranea	7.72621406691	Nakazawa et al [32]
OX_Smed_1.0.10743	Wnt2-1 mRNA, complete cds	Schmidtea mediterranea	7.10413299057	Petersen et al [16]
OX_Smed_1.0.22860	otxA mRNA, complete cds	Schmidtea mediterranea	3.23261657637	Martin-Duran et al [36]
OX_Smed_1.0.11668	Tryptophan hydroxylase (tph) mRNA, partial cds	Schmidtea mediterranea	3.05307067931	Fraguas et al [42]
OX_Smed_1.0.20218	Prohormone convertase 2 mRNA, complete cds	Schmidtea mediterranea	2.5238616996	Collins et al [29]
OX_Smed_1.0.11562	Eyes absent homolog 1	Homo sapiens	2.42655369488	Lapain et al [35]
OX_Smed_1.0.12486	Nuclear receptor TLX-1 (tlx-1) mRNA, complete cds	Schmidtea mediterranea	2.03263597349	Raska et al [43]
OX_Smed_1.0.08673	Sine oculis 1/2-2-like (Six1/2-2) mRNA, complete sequence	Schmidtea mediterranea	1.51454127627	Lapain et al [35]
OX_Smed_1.0.23033	Strain AAA-1 PREP homeodomain-like protein (prep) mRNA, complete cds	Schmidtea mediterranea	1.48015578861	Felix et al [22]

**Table 3:** **Tail enriched transcripts**

id	Blast hit	Species	Fold-change	Citation
OX_Smed_1.0.18076	wnt11-1 (wnt11-1) mRNA, complete cds	Schmidtea mediterranea	40.3156880815	Petersen et al [16]
OX_Smed_1.0.01500	Wnt11-2 mRNA, complete cds	Schmidtea mediterranea	32.2870501342	Petersen et al [16]
OX_Smed_1.0.07158	AbdBa Hox protein (abdba) mRNA, partial cds	Schmidtea mediterranea	14.3765686187	Iglesias et al [38]
OX_Smed_1.0.12463	HoxD-like protein (hoxD) mRNA, complete cds	Schmidtea mediterranea	11.4839009517	Martin-Duran et al [44]
OX_Smed_1.0.01988	Frizzled receptor-like protein (Frz-d) mRNA, partial cds	Schmidtea mediterranea	8.68234010817	Gurley et al [17]
OX_Smed_1.0.10882	wntP-2 (wntP-2) mRNA, complete cds	Schmidtea mediterranea	3.28341915342	Petersen et al [16]
OX_Smed_1.0.07540	wntP-3 (wntP-3) mRNA, complete cds	Schmidtea mediterranea	2.67985916157	Petersen et al [16]
OX_Smed_1.0.17841	Axis inhibition protein B (axinB) mRNA, complete cds	Schmidtea mediterranea	2.28993531882	Iglesias et al [39]
OX_Smed_1.0.04963	Marginal adhesive gland-1-like mRNA, partial sequence	Schmidtea mediterranea	2.21952894475	Zayas et al [45]
OX_Smed_1.0.11066	Evi/Wls mRNA, complete cds	Schmidtea mediterranea	2.11857383718	Adell et al [46]

**Table 4:** **Abbreviation of time-points and transcript lists**

Abbreviation	Description	Lists
**F**	Transcripts enriched in a head or tail fragment at 0 hours	**F-head** – transcripts enriched in head vs tail at 0 hours
		**F-tail** – transcripts enriched in tail vs head at 0 hours
**H**	Up and down-regulated transcripts in head fragments. Time-period is indicated after the abbreviation.	**H6-12-down** – Transcripts down-regulated from 6 to 12 hours in regenerating head fragments
		**H6-12-up** – Transcripts up-regulated from 6- 12 hours in regenerating head fragments.
**T**	Up and down-regulated transcripts in tail fragments. Time-period is indicated after the abbreviation.	**T36-48-down** – Transcripts down-regulated from 36-48 hours in regenerating tail fragments.
		**T36-48-up** – Transcripts up-regulated from 36-48 hours in regenerating tail fragments.
**O**	Up and down regulated shared transition between H and T.	**O-up** – Transcripts shared between H6-12-up and T36-48-up.
		**O-down** – Transcripts shared between H6-12-down and T36-48-down.
**P**	Transcripts up and down regulated in response to *smed-prep(RNAi)* tail fragment 24 hours after amputation	**P-up** – Transcripts down-regulated in smed-prep(RNAi) tail fragments 24 hours after amputation.
		**P-down** – Transcripts up-regulated in smed-prep(RNAi) tail fragments 24 hours after amputation.

**Figure 3:**
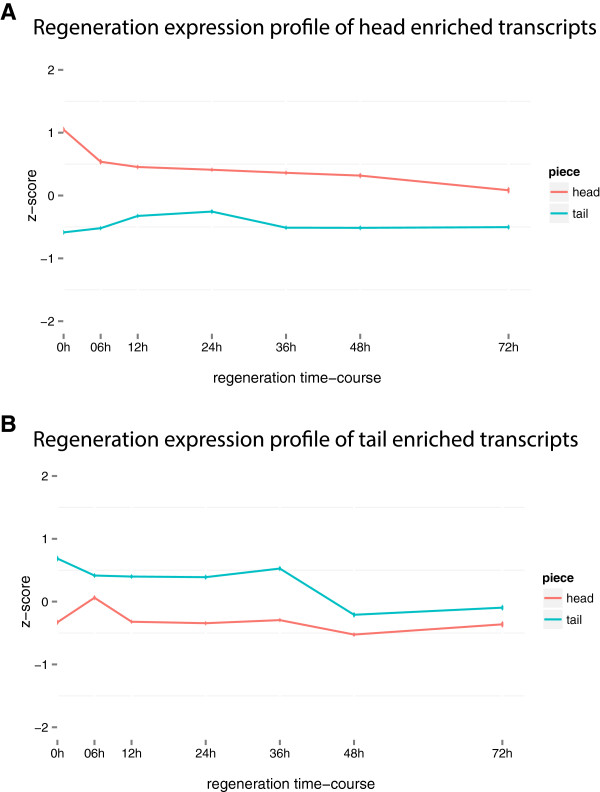
**Expression profiles of head and tail enriched transcripts. A)** The expression profile of 1,193 head enriched transcripts during the regeneration time-course. The x-axis displays the time-course and y-axis is a standardized expression value (z-score) calculated as the number of standard deviations away from the mean expression value for the specified transcript. The blue line represents tail time-course and red line, the head time-course. Head enriched transcripts are consistently up-expressed in head fragments compared to tail fragments. **B)** The expression profile of 1,289 tail enriched transcripts during the regeneration time-course. Expression of tail enriched transcripts converge at 48 hours between head and tail fragments.

We expected head and tail enriched transcripts to be representative of the existing anterior/posterior tissues. F-head and F-tail should be consistently up-regulated in their respective fragments until remodelling of the existing tissues occurs. We looked at the expression profile of these head and tail enriched transcripts during regeneration and found F-head transcripts to be consistently up-regulated in heads compared to tail fragments at the same time-points suggesting remodelling does not occur within the first 72 hours of head regeneration. In contrast, for tail enriched transcripts, there is a down-regulation in tail fragments at 48 hours (Figure 3) suggesting remodelling is taking place at this time.

There is also a slight up-regulation of head enriched transcripts during early tail regeneration that might reflect the development of the early brain structure shown previously to occur in anterior facing wounds [39, 47]. We found 277 transcripts out of a total 1,193 head enriched transcripts are up-regulated in tail fragments at 24 hours compared to 0 hours. Among these are several S. mediterranea transcripts involved in anterior development (*smed-prep*[22], *smed-ndk*[48], *smed-six*[34]) and transcripts known to be expressed anteriorly (*smed-wnt2-1*[37], *smed-sfrp*[41]) (Table 5). This group of 277 genes could be important in the early regeneration of anterior stuctures. Interestingly there are also several metalloproteinases (MMP) which have been implicated in neural tissue remodelling and cell migration in this list. However, early brain structures have previously been shown to develop in the blastema, not existing tissues and we also do not observe remodelling until 48 hours suggesting the function of MMPs at the stage is perhaps confined only to the blastema tissues or a small portion of the existing tissue.

**Table 5:** **Head enriched transcripts that are up-regulated in tail fragments from 0 hours to 24 hours**

ID	Description
OX_Smed_1.0.08673	sine oculis 1/2-2-like (Six1/2-2) mRNA
OX_Smed_1.0.17714	Smed-NDK-4 mRNA
OX_Smed_1.0.15141	secreted frizzled protein-like protein (SFRP-a) mRNA
OX_Smed_1.0.22718	Smed-NDK mRNA
OX_Smed_1.0.10743	Wnt2-1 mRNA
OX_Smed_1.0.23033	PREP homeodomain-like protein
OX_Smed_1.0.20161	Smed-NDK-3 mRNA
OX_Smed_1.0.08771	Neural proliferation differentiation and control protein 1
OX_Smed_1.0.22104, OX_Smed_1.0.09924, OX_Smed_1.0.20209, OX_Smed_1.0.21359	Zinc metalloproteinase nas-15
OX_Smed_1.0.00351, OX_Smed_1.0.03637, OX_Smed_1.0.22204	Matrix metalloproteinase
OX_Smed_1.0.12599, OX_Smed_1.0.23351, OX_Smed_1.0.18149	Synaptotagmin
OX_Smed_1.0.14894	Isoform C of Homeobox protein orthopedia

### A Shared regulatory program between head and tail regeneration

**Figure 4:**
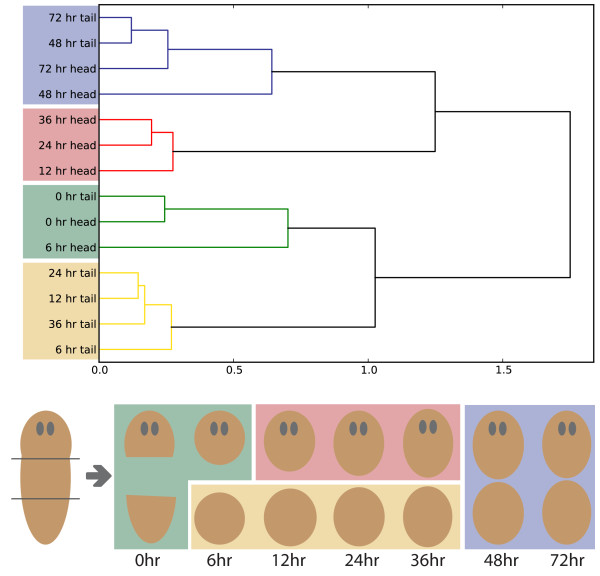
**Hierarchical clustering of sample libraries.** A hierarchical clustering performed using correlation distance and complete linkage was done on the filtered, normalized, and standardized counts for each library. The resulting clusters indicate a divergence of expression profiles between head and tail regeneration at 12 hours and a subsequent convergence of expression profiles at 48 hours.

We performed a hierarchical clustering of expression profiles using correlation distance on all libraries to observe when tail and head regeneration diverges (Figure 4). The clustering generated two separate groups of expression profiles (red, yellow) from 12 hours in heads and 6 hours in tails indicative of an early divergence in expression profiles. Surprisingly, we observed a subsequent convergence of expression profiles at 48 hours indicated by the blue cluster, grouping heads and tail fragments at 48 and 72 hours.

This convergence suggest both head and tail fragments reach a similar regenerative state at the transcriptome level as early as 48 hours. At this time point tails will have just formed cephalic ganglia tissue and started to form the beginnings of the photoreceptors [34, 47]. Nonetheless, we found this convergence surprising, as head and tail fragments are still morphologically distinct at this early stage. Together this suggested to us that much of the similarity of expression levels might represent underlying gene regulatory and cellular behaviours rather than the formation of equivalent tissues.

**Figure 5:**
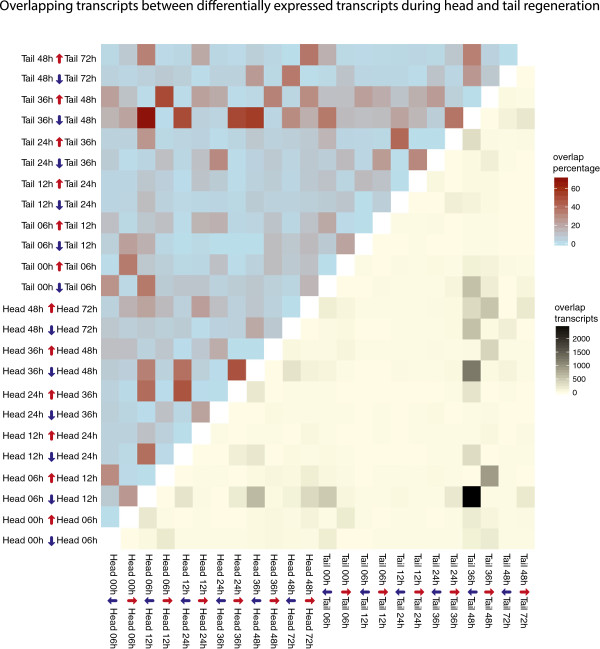
**Overlapping transcripts between sets of differentially expressed transcripts.** A heatmap displaying the number of overlapping transcripts between various differential expression lists. Differential expression is defined here as p-value < 0.01 and fold-change > 2. Both upper left and lower right triangles display the same information, but in different metrics. The upper left triangle (red to blue gradient) shows the percentage overlaps between two differential expression lists. Percentage overlap is calculated as the average of the two overlap percentages generated by dividing number of share transcripts to each of the two differential expression list totals. The lower right triangle (yellow to black) shows the absolute numbers of overlaps between two differential expression lists.

**Figure 6:**
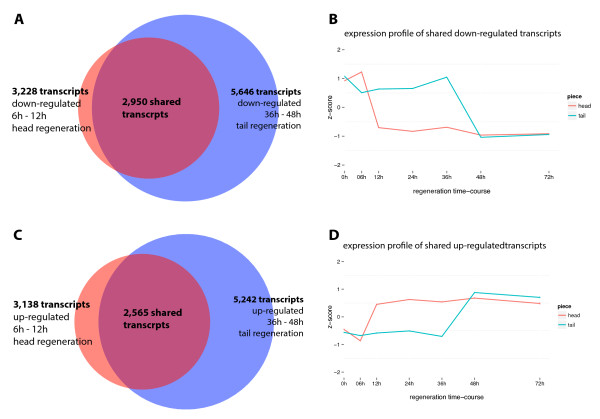
**Shared transcripts between H6-12 and T36-48. A)** The number of shared transcripts between down-regulated transcripts in tails from 36-48 hours and down-regulated transcripts in heads from 6 to 12 hours. **B)** The expression profile of the shared transcripts between H6-12 and T36-48 down-regulation during regeneration showing the early divergence caused by head undergoing this program and subsequent convergence caused by tail going through the same program. The x-axis displays the time-course and y-axis is a standardized expression value (z-score) calculated as the number of standard deviations away from the mean expression value for the specified transcript. **C)** The number of shared transcripts between up-regulated transcripts in tails from 36-48 hours and up-regulated transcripts in heads from 6 to 12 hours. **D)** The expression profile of the shared transcripts between H6-12 and T36-48 up-regulation during regeneration.

To investigate the convergence further we looked for shared batteries of genes between differentially regulated genes at each time point (Figure 5). The most significant overlapping regulatory programs with a hypergeometric p-value of effectively 0 were observed for 2 differentially expressed lists: H6-12-down and T36-48-down (Figure 6A), H6-12-up and T36-48-up (Figure 6C). We conclude that the H6-12 regulatory transition is primarily responsible for the early divergence of expression profiles between head and tail fragments and the subsequent convergence at 48 hours is caused by tails going through a very similar regulatory transition during T36-48 (Figure 6B and D). We will refer to the shared regulatory program between H6-12 and T36-48 as O and we will refer the shared up/down-regulation program as O-up and O-down.

O-up and O-down both represent approximately one third of transcripts assessed for differential expression. We found 69 potential transcription factors out of a total 467 potential transcription factors in our data set were present in O-down and 29 in O-up. Known *S. mediterranea* transcript factors found in O-down include *smed-prep*, *smed-dlx*, *smed-gata*, *smed-prox1*, and *smed-six*. In O-up, we found *smed-junli, smed-tcf15, smed-e2f-like*.

**Figure 7:**
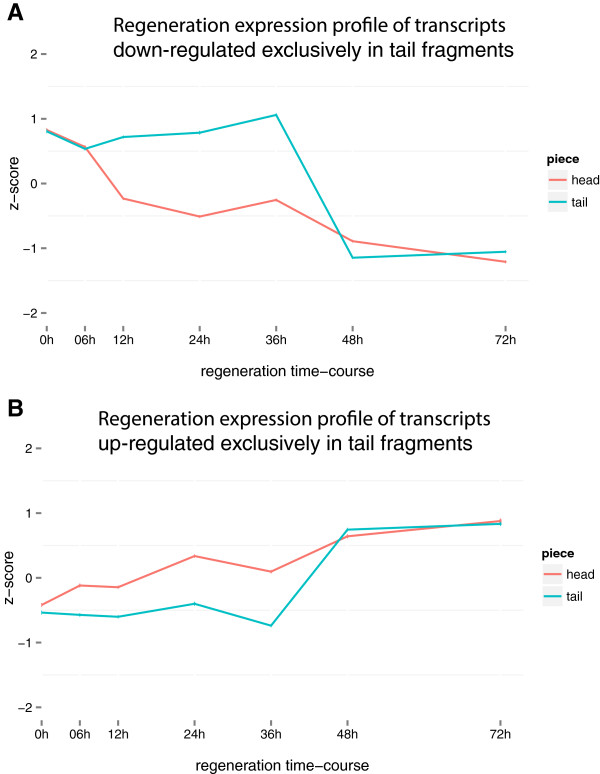
**Expression profiles of transcripts exclusively regulated in T36-48. A)** The expression profile of 2,696 transcripts that are down-regulated exclusively during T36-48. The x-axis displays the time-course and y-axis is a standardized expression value (z-score) calculated as the number of standard deviations away from the mean expression value for the specified transcript. **B)** The expression profile of 2,677 transcripts that are up-regulated exclusively during T36-48.

There are more transcripts up/down-regulated in T36-48 compared to H6-12. 91% of the transcripts in H6-12-down overlap with 52% of transcripts in T36-48-down and 81.7% of H6-12-up transcripts overlap with 49% of T36-48-up transcripts meaning there are 2,696 and 2,677 transcripts that are exclusively regulated (not shared with H6-12) in T36-48-down and T36-48-up. While these transcripts were not found to be differentially expressed at H6-12, they still conform to the convergence of expression profile at 48 hours (Figure 7). Instead of being sharply regulated during H6-12, these transcripts gradually up/down-regulate during head regeneration to eventually match tail expression level at 48 hours.

Together our analysis reveals a previously unknown shared regulatory program between two very different regenerative scenarios. This program includes a large proportion of the genome and runs at different times and scenarios. This gives us for the first time insight into how whole body regeneration is regulated and suggests that different regenerative scenarios may use a core regenerative program that is activated after scenario specific events have occurred. Future work investigating an even wider set of regenerative scenarios will test this model, but the use of shared program between the opposite scenarios investigates here is strongly suggestive this is the case. This program is likely to represent key shared events, such as elaboration of axial fates, replacement of major tissues such as the gut [49], excretory system and nervous system and re-establishment of the stem cell and stem cell progeny populations [50], and remodelling of existing tissues to their correct proportions.

### Smed-prep(RNAi) disrupts the expression of transcripts found in the two major regulatory events during regeneration

*Smed-prep* is a TALE class homeodomain gene that has been found to be required for anterior fate and patterning during regeneration [22]. Upon RNAi knock-down of *smed-prep*, regenerating tail fragments fail to develop a discernible anterior compartment. In order to provide a genome wide validation of our time-course dataset and to investigate possible down-stream genes regulated directly or indirectly by *smed-prep*, we performed RNA-seq on tail fragments of *smed-prep(RNAi)* animals 24 hours after amputation along with GFP dsRNA injected controls at the same time-point. We will refer to this 24 hour tail fragment comparison between gfp and *smed-prep(RNAi)* animals as P. Transcripts down and up regulated in response to *smed-prep(RNAi)* will be referred to as P-down and P-up.

We generated two lists of differentially expressed transcripts for P-down and P-up at a fold-change of 2 or more. There are 1,236 transcripts in P-up and 591 in P-down. The larger number of transcripts up-regulated in response to *smed-prep(RNAi)* versus down-regulation suggest a direct or indirect transcriptional repressive role of *smed-prep*.

As a validation of our *smed-prep(RNAi)* data, we looked at whether F-head transcripts are affected by *smed-prep(RNAi*). We found 51 F-head transcripts in P-down and 47 in P-up. While there are some voltage gated channels and neurotransmitter transcripts in these lists, no known *S. mediterranea* transcripts were found and only two transcription factors were found in both P-down and F-head (an achaete-scute homolog and zinc finger protein). This result was to be expected as F-head transcripts are not up-regulated in tail fragments during the regeneration time-course. There was also no significant enrichment of the F-head transcripts that were up-regulated in tail regeneration at 24 hours in either P-down or P-up. This suggests that *smed-prep* is not involved in early brain development, in agreement with previous work that has investigated early brain regeneration [47].

**Figure 8:**
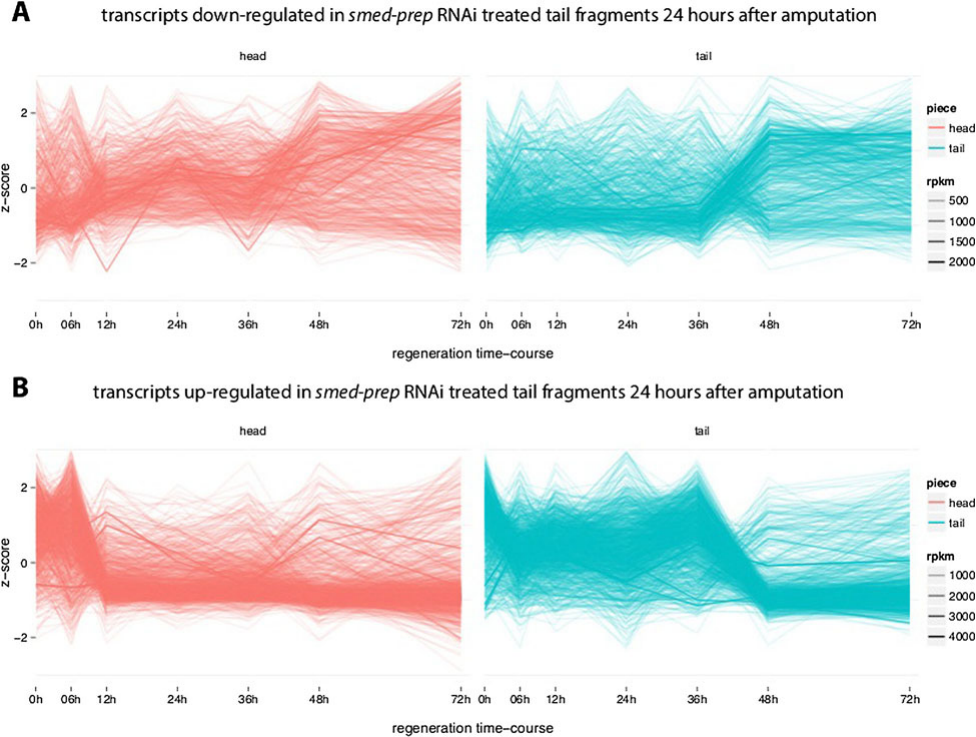
**Head and tail regeneration profile of** ***smed-prep*** **RNAi response transcripts. A)** The expression profile of transcripts down-regulated in response to *smed-prep* RNAi in tail fragments 24 hours after amputation. The x-axis displays the time-course and y-axis is a standardized expression value (z-score) calculated as the number of standard deviations away from the mean expression value for the specified transcript. Each line represents the expression profile of a single transcript and the opacity of the line represents the RPKM value. **B)** The expression profile of transcripts up-regulated in response to *smed-prep* RNAi in tail fragments 24 hours after amputation.

We looked at the expression profiles of P during regeneration and found that 330 P-down transcripts are in T36-48-up and 878 P-up transcripts are in T36-48-down (Figure 8) with significant enrichment (5.5e-41 and 3.4e-185). While P-up overlapped significantly with O-down, P-down overlapped with transcripts up-regulated in T36-48 exclusively (and not in H6-12-up) (Figure 8). The P-up data is showing that *smed-prep* is possibly playing a direct or indirect repressive role which facilitates O-down. Since the phenotype of *smed-prep(RNAi)* is loss of the anterior structures, we can reasonably assume that some of P-down is involved in anterior development. The observation that P-down seem to only relate to T36-48-up exclusively suggest T36-48 is when smed-prep is up-regulating these anterior developmental transcripts; whereas it was not necessary to do so in head fragments since anterior structures already exists.

The expression profile of P-up and P-down during regeneration allowed us to observe the differential role of *smed-prep* in both head and tail fragments. In head fragments, *smed-prep* plays a repressive role during the early major regulatory transition in heads. In tail fragments, in addition to also playing the same repressive role during the later major regulatory transition, it activates the anterior structure regeneration program.

### Triclad specific transcripts are enriched in differentially expressed transcripts during regeneration

We annotated the consolidated transcriptome by blasting against 12 proteomes*: Caenorhabditis elegans, Drosophila melanogaster, Danio rerio, Homo sapiens, Mus musculus, Schistosoma mansoni, Clonorchis sinensis, Strongylocentrotus purpuratus, Nematostella vectensis Lottia gigantea, Helobdella robusta, Capitella teleta* and 3 transcriptomes: *Girardia trigrina, Procotyla fluviatilis*[51]*, Denrocoelum lacteum*[52]. At an e-value threshold of 1e-5 or less, 20,603 transcripts had at least one hit and 19,478 transcripts at e-value threshold of 1e-15 or less.

**Figure 9:**
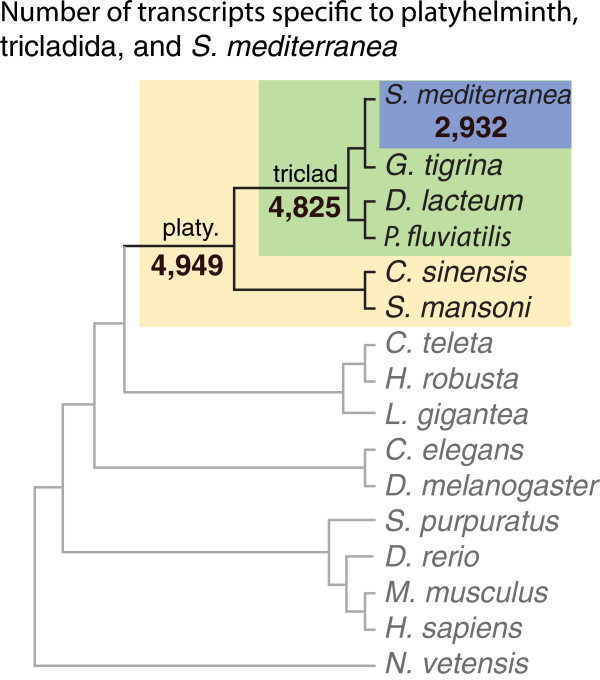
**Transcripts specific to platyhelminth, tricladida, and** ***S. mediterranea*** **.** 15 proteomes/transcriptomes across the animal kingdom were used to blast against the consolidated transcriptome. Several different e-value thresholds were used to generate lists of platyhelminth, tricladida, and *S. mediterranea* specific transcripts.

We categorized the transcripts based on species hits to generate lists of transcripts that were potentially *S. mediterranea* specific, triclad specific, and platyhelminth specific (Figure 9). We performed this analysis on increasing e-value strictness and found 2,932 potentially *S. mediterranea* specific transcripts, 4,825 triclad specific transcripts, and 4,949 platyhelminthes specific transcripts at the highest strictness level (Table 6).

**Table 6:** **Platyhelminth, triclad, and S. mediterranea specific transcripts**

S. mediterranea specifc	No hits at e-value < 1e-5: 2,942	No hits at e-value < 1e-10: 2,942	No hits at e-value < 1e-15: 2,942
**Triclad specific**	No hit to non-triclads at e-value < 1e-5 and hit to only triclads at e-value < 1e-5: **6,441**	No hit to non-triclads at e-value < 1e-5 and hit to only triclads < 1e-10: **5,393**	No hit to non-triclads at e-value < 1e-5 and hit to only triclads < 1e-15: **4,825**
**Platyhelminthes specific**	No hit to non-platyelminth at e-value < 1e-5 and hit to only platyelminthes at e-value < 1e-5: **6,912**	No hit to non-platyelminth at e-value < 1e-5 and hit to only platyelminthes at e-value < 1e-10: **5,594**	No hit to non-platyelminth at e-value < 1e-5 and hit to only platyelminthes at e-value < 1e-15: **4,949**

In addition to blast annotations, we also performed protein domain predictions on the transcriptome using models from the PFAM database [53] resulting in 13,217 transcripts with domain annotations. Using this domain information, we were able to look at the composition of domains within the strict platyhelminth and triclad specific lists of transcripts.

Within the platyhelminthes specific transcripts, 289 transcripts had pfam annotations. The low number of domain annotations probably reflects the heavy bias of known protein domains towards nematode, insect and vertebrate systems. We found that the most abundantly represented domain in platyhelminth specific list was the 7 transmembrane receptor domain (7tm) of the rhodopsin family (PF00001) found in 13 transcripts. This agrees with a previous study which catalogued the repertoire of G protein-coupled receptors (GPCR) in *S. mediterranea* and found a large expansion of platyhelminth specific GPCR of the rhodopsin subfamily [54].

Within the triclad specific transcripts, 262 transcripts had pfam annotations with the ubiquitin domain (PF00240) being most represented in 12 transcripts. The ubiquitin protease system (UPS) is the main cellular proteolytic mechanism that uses ubiquitin to tag and target proteins for degradation. Several studies have implicated UPS in drosophila development [55] and regeneration in various systems [56–58] making UPS a potential target for further research.

**Figure 10:**
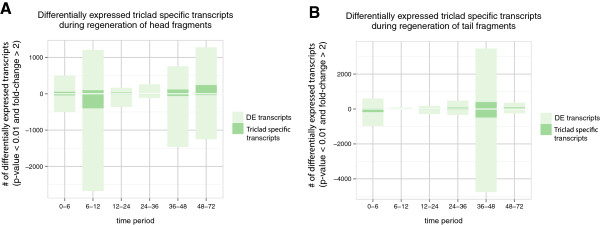
**Enrichment of triclad specific transcripts during head and tail regeneration. A)** The number of triclad specific transcripts that are differentially expressed during head regeneration. **B)** The number of triclad specific transcripts that are differentially expressed during tail regeneration.

We also looked at the expression profile of triclad specific transcripts during regeneration and found there was significant enrichment in differentially expressed transcripts (p-value < 0.01 and fold-change > 2) during both head and tail regeneration (Figure 10, Tables 7 and 8).

**Table 7:** **Enrichment p-values of S. mediterranea specific transcripts during head regeneration**

	0-6 hours	6-12 hours	12-24 hours	24-36 hours	36-48 hours	48-72 hours
**Up-regulation**	1	1	1	1	1.1e-6	5.2e-17
**Down-regulation**	1	6.3e-18	1	1	1	1

**Table 8:** **Enrichment p-values of S. mediterranea specific transcripts during tail regeneration**

	0-6 hours	6-12 hours	12-24 hours	24-36 hours	36-48 hours	48-72 hours
**Up-regulation**	1	1	1	1	2.8e-7	1e-11
**Down-regulation**	2.6e-14	1	1	1	1	1

Together our data identify a large set of potentially novel and/or rapidly evolving genes that are clearly differentially expressed during regeneration. Previous studies in planarians and other regenerative models have highlighted a role of conserved genes, known from studies of development, as key regulators of regeneration. More recent genome wide studies using transcriptomics have revealed that lineage specific genes may also be important and require study [24, 25]. Our data also support that this may be the case during planarian regeneration. In particular we uncover an enrichment of novel genes during a regulatory transition that is shared between different regenerative scenarios. Our data suggest that the potent regenerative capacity in planarians may be partly due to novel mechanisms conserved within the highly regenerative triclad clade and pave the way for functional study of these genes.

## Conclusion

In this study we have generated a consolidated transcriptome from 5 independently assembled transcriptomes and available ESTs. This consolidated dataset represents a high confidence set of transcripts providing a valuable resource for future expression studies. Our regeneration transcriptome consisting of regenerating head and tail fragments from 0 to 3 days reveals a shared regulatory program consisting of over 5,000 transcripts active at temporally shifted time-periods between regenerating head (6-12 hours) and in tail fragments (36-48 hours). Additional RNA-seq experiments on *smed-prep(RNAi)* animals versus control tail fragments allowed us to find transcripts that are regulated differentially in response to *smed-prep*. We observed that these *smed-prep* response transcripts are enriched during the shared regulatory program during regeneration suggesting an involvement in brain regeneration. We also performed BLAST alignment to 15 species across eukaryotes to identify novel or divergent genes and found lists of *S. mediterranea*, triclad, and platyhelminth specific transcripts. Triclad specific transcripts are found to be enriched in differentially expressed transcripts throughout regeneration suggesting novel mechanisms may contribute to the animal’s potent regenerative capacity.

## Methods

### Animal culture

A clonal line of the asexual strain of Schmidtea mediterranea, AAANOTBIOL01, was used for all experiments. Animals were reared at 20°C in tap water filtered through activated charcoal and buffered with 0.5 ml/L 1 M NaHCO3. Planarians were fed veal liver and starved for at least one week prior to experiments or amputation. All worms used were 7–8 mm in length. The animals used in these experiments do not require approval from the ethical committee.

### Smed-prep(RNAi) for RNAseq

RNAi for *Smed-prep* was perfomed by two rounds of injection as previously described [22].

### Preparation of RNA form a regenerating timecourse

Regenerating head and tail fragments from 20 worms at each timepoint were collected and snap frozen at 6, 12, 24, 36, 48, and 72 hours of anterior and posterior regeneration in two replicate samples for total RNA preparation (Trizol). RNA was also prepared from *Smed-prep(RNAi)* regenerating tails at 24 hours. Total RNA was also prepared and pooled from a regenerative time course of a sexual strain of *G. tigrina*.

### Library preparation and sequencing

RNA from regenerative stages was enriched for mRNA enriched using the Poly A Purist Kit (Ambion, Cat. No. AM1919) followed by further depletion of ribosomal RNA using the Ribominus Eukaryotic kit (Invitrogen, Cat. No. A10837-08). Solid whole transcriptome libraries were made as outlined in the Solid Whole transcriptome kit protocol (Applied Biosystems, Cat. No. 4425680). The Quant-it HS dsDNA assay kit (Invitrogen, Cat. No. Q32851) was used to measure the concentration of libraries. Sequencing was performed on a SOLiD 3 ABi sequencer according to the manufacturer’s instructions to generate 50 bp reads in colour space. All data is available at the NCBI short read archive under study number SRP002478.

454 sequencing and assembly was performed from *G. tigrina* total RNA as previously described [20].

### Transcriptome consolidation

Detailed information on the transcriptome consolidation process is included in the Additional files 1, 2, 3, 4, 5, 6, 7, 8.

### Transcriptome annotation

BLASTX alignment was performed against *Caenorhabditis elegans, Drosophila melanogaster, Danio rerio, Homo sapiens, Mus musculus, Schistosoma mansoni, Clonorchis sinensis* proteomes and TBLASTX was performed against *Girardia tigrina* transcriptome*.* A constant database size of total base pairs of all sequences was used to ensure comparable e-values. PFAM annotations was performed with HMMScan using the PFAM-A database. The gathering cut-off threshold was used for HMMScan. Transcription factors were identified using manually curated PFAM domain profiles from DBD: Transcription factor prediction database.

### Read mapping and differential expression analysis

Reads were mapped with the ABI LifeScope software’s single fragment mapping module. Uniquely mapped reads were counted for each transcript on both strands with HTSeq-count [59] using a mapping quality filter of 30. Outliers were filtered out by removing transcripts with tag counts that were more than 1% of the total library in at least 3 sequenced libraries. Transcripts with less than 20 reads in all libraries were also removed. Normalized counts and differential analysis was performed with EdgeR [31]. The generalized linear model function of edgeR was used across all the libraries.

Read mapping and tag counting for *smed-prep* (RNAi) RNAseq samples was performed the same way as the regeneration time-course. Outliers were also removed based on more than 1% of total reads in at least 2 libraries. Transcripts with less than 50 reads were removed from all libraries. Since there were no replicates for these samples, we did not use edgeR to calculate differential expression as EdgeR requires replicates to effectively model the dispersion among libraries of the same conditions. We instead relied on a high filter of 50 reads for lowly expressed transcripts and a fold-change threshold of at least 2 for assessing differential expression.

### Availability of supporting data

The raw sequencing data was deposited into short read archive with these two study accession numbers: PRJEB4680 (G. tigrina raw Roche 454 data), PRJEB4686 (S. mediterranea regeneration time-course ABI SOLiD data). The transcriptome data for both S. mediterranea and G. tigrina was uploaded to FigShare at this address and DOI: Aboobaker Lab Schmidtea mediterranea transcriptome dataset. Damian Kao. figshare. http://dx.doi.org/10.6084/m9.figshare.801077. Additionally, this data is incorporated into PlanMine (http://planmine.mpi-cbg.de/).

## Electronic supplementary material

Additional file 1: **ZIP file containing a .pdf document of detailed methods including how consolidation was performed and various analysis.** Also includes relevant python scripts for each step of the methods. (ZIP 198 KB)

Additional file 2: **FASTA file of consolidated transcripts.** (ZIP 11 MB)

Additional file 3: **ZIP file containing FASTA file of differentially expressed transcripts in H6-12-down, H6-12-up, T36-48-down, T36-48-up, F-head, F-tail, O-down, O-up, P-down, P-up, F-head + T0-24-up.** Also contains platyhelminth, triclad, and *S. mediterranea* specific transcripts. (ZIP 18 MB)

Additional file 4: **Tab delimited file of filtered and normalized tag counts for the regeneration time-course.** (ZIP 3 MB)

Additional file 5: **Tab delimited blast annotations against selected proteomes for consolidated transcriptome.** (ZIP 2 MB)

Additional file 6: **Tab delimited gene ontology annotations for consolidated transcriptome.** (ZIP 76 KB)

Additional file 7: **Tab delimited file of filtered and normalized tag counts for smed-prep RNAi dataset.** (ZIP 168 KB)

Additional file 8: **FASTA file of** ***G. tigrina*** **transcriptome.** (ZIP 9 MB)
