# Supplementary material for: The planarian regeneration transcriptome reveals a shared but temporally shifted regulatory program between opposing head and tail scenarios
Source: BMC Genomics. 2013 Nov 16;14(1):797. doi: 10.1186/1471-2164-14-797 (PMC4046745; doi:10.1186/1471-2164-14-797)
Supplement: Supplementary file 1 — Additional file 1: ZIP file containing a .pdf document of detailed methods including how consolidation was performed and various analysis. Also includes relevant python scripts for each step of the methods. (ZIP 198 KB) [file 12864_2013_5532_MOESM1_ESM.zip › consolidation.pdf]

Transcriptome consolidation process
All scripts used in the consolidation process are included in the supplemental data. Alternatively a github page is available at:
https://github.com/damiankao/planarian-regeneration-transcriptomics with the scripts. All scripts are commented specifying input formats and prerequisites.

The consolidation process included 5 transcriptome datasets and 1 EST dataset. Below are some general length metrics on the consolidated transcriptome and the 6 input datasets.

Transcript lengths

	**mean**	**median**	**N50**
**Aboobaker**	950.52	804	1169
**Bartscherer**	1074.91	715	1739
**Rajewsky**	1019.57	807	1261
**Graveley**	931.22	685	1323
**Pearson**	921.61	595	1299
**ESTs**	620.38	655	673
**Consolidated**	1738.43	1411	2126

There are 6 main steps in the consolidation process. This document will describe these steps in detail.

1. Supplemental data generation. Several supplemental data files need to be generated for the later steps of the consolidation process. These include: blasting the 5 transcriptomes against selected proteomes, generating transcript nucleotide lengths for each transcriptome, generating longest open reading frame lengths for each transcriptome.

8 proteomes were used for blast: *C. elegans, D. melanogaster, H. sapiens, M. musculus, D. rerio, S. purpuratus, S. mansoni, C. sinensis*. The following blast command was used:

blastx -query transcriptome_smed_consolidated.renamed.fa -db SPECIES -evalue 1e-15 -outfmt ‘6 qseqid qlen sseqid slen qstart qend sstart send evalue bitscore qframe’ -max_target_seqs 20 -dbsize 150000000

The -dbsize flag was used to make sure e-values among blasts were comparable.

fa_getLengths.py script was used to get the nucleotide length. For obtaining the longest ORF length, the EMBOSS (http://emboss.sourceforge.net/) getORF tool was used on default settings

where an amino acid sequences between stop codons were extracted. fa_longestORF.py was then used to parse the resulting file to generate a .fasta file of longest ORF sequence. The previous fa_getLengths.py script was used on the longest ORF .fasta file to finally get the lengths.

2. Sequence clustering. All input datasets (5 transcriptome, 1 EST) .fasta files was first renamed for easier parsing and downstream analysis. The new .fasta entry header is simply the last name of the principal investigator and an incremented number delimited by a period, ie. aboobaker.1, bartscherer.51, est.8.

An initial clustering of the the input datasets was performed using CAP3 on 97% identity threshold. The following command was used:

cap3 transcriptome_smed_allDatasets.renamed.fa -p 97

The resulting output file (stdout output) was parsed with consolidated_parseCAP3.py generating a cluster file where the first column is the assembled contig and the second column is a comma separated list of member transcripts that made up the assembled contig.

The 5 transcriptome and 1 EST datasets produced 26,004 clusters total. The contributing data sources for these clusters are as follows:

	**Number of contributing transcripts**	**Number of clusters with transcripts**
**Aboobaker**	17,308	15,070
**Bartscherer**	15,037	12,384
**Rajewsky**	19,948	15,437
**Graveley**	10,780	8,133
**Pearson**	16,949	15,242
**ESTs**	69,449	13,339

consolidated_multiSource.py was used to extract clusters that contained members from at least 2 data sources. 23,802 clusters remained and are used for the next step.

3. Resolving fusion events. A fusion score is calculated for every transcript using the blast results from step1. Fusion score is the length of the longest blast alignment divided by the length of all collapsed blast alignments. This method will only detect potential fusion transcripts where the all fused members have blast homology.

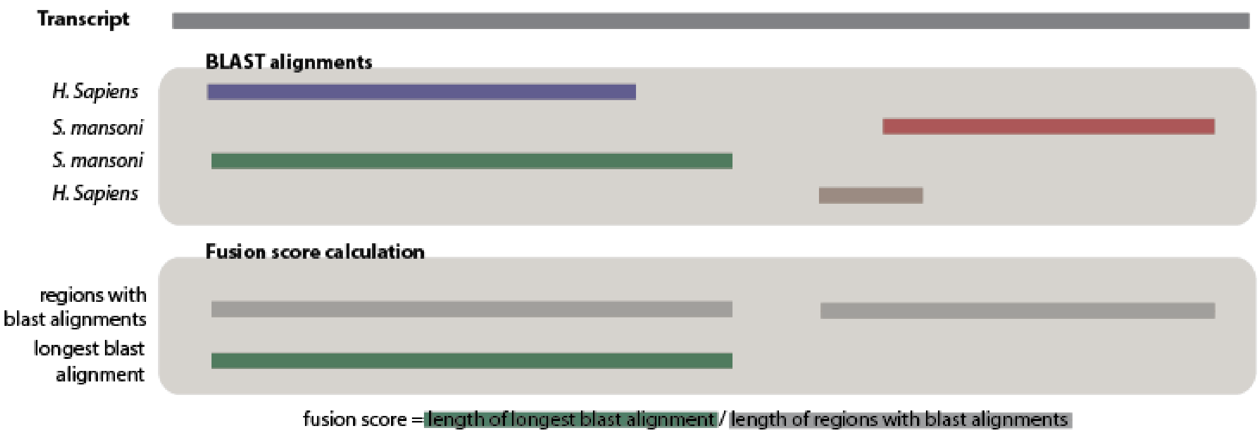

consolidated_fusionScore.py was used on the blast results to calculate a fusion score for each transcript. This script generates a tab delimited file with the following columns: transcript id, fusion score, coordinates of the longest blast hit, coordinates of all regions with blast hits.

From the 5 transcriptome datasets, 728 transcripts with fusion score <= 0.6 were extracted as potential fusion transcripts. Among these, 55 were from Aboobaker, 168 from Bartscherer, 47 from Graveley, 378 from Pearson, and 80 from Rajewsky.

All clusters that contain a potential fusion member were extracted. Out of 23,802 clusters generated in step 2, 441 clusters contained fusion members. Potential fusion members were removed from these clusters. A .fasta file of remaining non-fusion member sequences for each cluster was generated.

These 441 clusters with potential fusion transcripts taken out were re-clustered with cap3 using the same command in step 1. The resulting assembled contigs and singlets were added back to the original cluster file. 441 clusters with at least one fusion member was separated into 1,014 clusters/singlets.

4. Retain transcript with the longest open read frame. The sequence data associated with each cluster are the member transcripts and the cap3 assembled contig. The assembled contig is considered the representative of each cluster.

Sequence errors in any of the individual member sequences might be reproduced in the assembled cap3 contig resulting in a truncated ORF. In cases where a member transcripts that are similar in length to the cap3 assembled contig and has a longer ORF exists, we want to retain that transcript.

Using the nucleotide length and longest ORF length data gathered in step 1, we use consolidated_getRep.py script to get the sequence with the longest ORF within each cluster.

This script will first, for each cluster, filter out member transcripts that are less than 90% length of the assembled contig. Then from the remaining sequences (including the assembled contig), pick the sequence with the longest ORF.

This process resulted in 2,487 clusters using a member transcript as the longest ORF sequence.

5. Missing known S. mediterranea mRNA. There are around 1,300 S. mediterranea mRNA sequences deposited at the NCBI database. Most of the sequences are partial sequences and unknown clones. 179 complete mRNA sequences were taken from this mRNA set to check the coverage of the consolidated transcriptome. 16 mRNAs were found not to be in the consolidated transcriptome:

neuropeptide Y prohormone-11
neuropeptide Y prohormone-10
secreted peptide prohormone-3
neuropeptide Y prohormone-8
neuro peptide precursor12
secreted peptide prohormone7
secreted peptide prohormone8
bcl2-2
C3H-zinc finger-containingprotein1
cdk1-like protein
cyclinB-like protein
cdk2-like protein
GLI-2 (gli-2)
noggin-like protein6 (nlg6)
noggin-like protein4 (nlg4)
wntP-1 (wntP-1)

We added these back into the consolidated transcriptome for a final .fasta file containing 23,545 transcript sequences.

Transcriptome tag count
Tag counting was performed with HTSeq using this command:

samtools view mapped.bam | htseq-count -a 30 transcripts.gtf

A strict mapping quality of 30 was used for all libraries. A low tag count filter was performed on the regeneration tag count data which removed any transcript that had less than 20 counts in all libraries (counts_filterLow.py). An outlier filter (counts_topExpression.py) was also performed to remove any transcript that made up 1% or more of the total library tag counts in more than 3 libraries (OX_Smed_1.0.07173, OX_Smed_1.0.10171, OX_Smed_1.0.21787,

OX_Smed_1.0.03228, OX_Smed_1.0.07610, OX_Smed_1.0.17315, OX_Smed_1.0.02939, OX_Smed_1.0.19392. These 8 outliers included heat shock protein 90, cytochrome oxidase, translation elongation factor, cathepsin, mitochondrial solute carriers, glutathione transferase and 2 without blast annotations.

Differential expression analysis
EdgeR was used to perform differential expression analysis (de_edger.R).

Hierarchical clustering of libraries
Hierarchical clustering and plotting was done with python using the scipy and matplotlib libraries (cluster_hierarchical.py). Correlation distance and complete linkage was used on filtered, edgeR normalized, and standardized tag counts.

Clade specific transcripts
We generated platyhelminth, triclad and S. mediterranea specific transcripts by analyzing blast result of the consolidated transcriptome against 15 other species (*Caenorhabditis elegans, Drosophila melanogaster, Danio rerio, Homo sapiens, Mus musculus, Schistosoma mansoni, Clonorchis sinensis, Strongylocentrotus purpuratus, nematostella vectensis Lottia gigantea, Helobdella robusta, capitella teleta*, *Girardia trigrina, Proctotyla fluviatilis Denrocoelum lacteum*).

We used three levels of strictness based on e-values.
S. mediterranea specific: Transcripts that do have have any blast hits at e-values less than 1e-5, 1e-10, 1e-15.
Triclad specific: Transcripts that blast to any combination of *G. tigrina, P. fluviatilis, D. lacteum* at e-value of 1-e15, 1e-10, 1e-5 or less and blast to other species at e-value more than 1e-5; with the addition of *S. mediterranea* specific transcripts at 1e-5.
Platyhelminth specific: Transcripts that blast to any combination of *C. sinensis, S. mansoni, G. tigrina, P. fluviatilis, D. lacteum* at e-value of 1e-15, 1e-10, 1e-5 or less and blast to other species at e-value more than 1e-5; with the addition of *S. mediterranea* specific transcripts at 1e-5.
